# *D4Z4* Methylation Levels Combined with a Machine Learning Pipeline Highlight Single CpG Sites as Discriminating Biomarkers for FSHD Patients

**DOI:** 10.3390/cells11244114

**Published:** 2022-12-18

**Authors:** Valerio Caputo, Domenica Megalizzi, Carlo Fabrizio, Andrea Termine, Luca Colantoni, Cristina Bax, Juliette Gimenez, Mauro Monforte, Giorgio Tasca, Enzo Ricci, Carlo Caltagirone, Emiliano Giardina, Raffaella Cascella, Claudia Strafella

**Affiliations:** 1 Genomic Medicine Laboratory-UILDM, Santa Lucia Foundation IRCCS, 00179 Rome, Italy; 2 Department of Biomedicine and Prevention, Tor Vergata University, 00133 Rome, Italy; 3 Data Science Unit, Santa Lucia Foundation IRCCS, 00179 Rome, Italy; 4 Epigenetics and Genome Reprogramming Laboratory, Santa Lucia Foundation IRCCS, 00179 Rome, Italy; 5 Unità Operativa Complessa di Neurologia, Fondazione Policlinico Universitario A. Gemelli IRCCS, 00168 Rome, Italy; 6 John Walton Muscular Dystrophy Research Centre, Newcastle University and Newcastle Hospitals NHS Foundation Trusts, Newcastle Upon Tyne NE1 3BZ, UK; 7 Istituto di Neurologia, Università Cattolica del Sacro Cuore, 00168 Rome, Italy; 8 Department of Clinical and Behavorial Neurology, Santa Lucia Foundation IRCCS, 00179 Rome, Italy

**Keywords:** FSHD, epigenetics, DNA methylation, neuromuscular diseases, biomarker, machine learning, *D4Z4*

## Abstract

The study describes a protocol for methylation analysis integrated with Machine Learning (ML) algorithms developed to classify Facio-Scapulo-Humeral Dystrophy (FSHD) subjects. The DNA methylation levels of two *D4Z4* regions (DR1 and *DUX4*-PAS) were assessed by an in-house protocol based on bisulfite sequencing and capillary electrophoresis, followed by statistical and ML analyses. The study involved two independent cohorts, namely a training group of 133 patients with clinical signs of FSHD and 150 healthy controls (CTRL) and a testing set of 27 FSHD patients and 25 CTRL. As expected, FSHD patients showed significantly reduced methylation levels compared to CTRL. We utilized single CpG sites to develop a ML pipeline able to discriminate FSHD subjects. The model identified four CpGs sites as the most relevant for the discrimination of FSHD subjects and showed high metrics values (accuracy: 0.94, sensitivity: 0.93, specificity: 0.96). Two additional models were developed to differentiate patients with lower *D4Z4* size and patients who might carry pathogenic variants in FSHD genes, respectively. Overall, the present model enables an accurate classification of FSHD patients, providing additional evidence for DNA methylation as a powerful disease biomarker that could be employed for prioritizing subjects to be tested for FSHD.

## 1. Introduction

Facio-Scapulo-Humeral muscular Dystrophy (FSHD) is caused by an aberrant expression of *DUX4* that results from a partial reduction of the Repeated Units (RU) located in the subtelomeric *D4Z4* macroarray (4q35). Generally, healthy individuals display *D4Z4* size ranging from 11 to 100 RU, in contrast to the 1 to 10 RU (namely, *D4Z4* reduced allele or DRA) observed in FSHD1 subjects. In addition, the presence of subtelomeric variants of the 4q (namely, 4qA or permissive allele) have been associated with FSHD [1]. Furthermore, detrimental variants in *SMCHD1*, *LRIF1* and *DNMT3B* have been described as causative genes (i.e., FSHD2) or disease modifiers with or without the presence of DRA [1,2,3,4,5,6,7,8,9]. Moreover, the above-mentioned genetic alterations were associated with epigenetic changes at the *D4Z4* locus, such as DNA hypomethylation that has been reported to contribute to FSHD [1,10]. Despite the current knowledge concerning the molecular mechanisms of disease, the variable expressivity and incomplete penetrance of FSHD complicate and delay the time for a proper diagnosis, clinical care, and follow-up of affected patients. To date, the molecular diagnosis is still based on the detection of DRAs by means of Linear- or Pulsed-Field Gel Electrophoresis (PFGE) and Southern Blotting. Next Generation Sequencing (NGS) and direct resequencing are usually performed to detect pathogenic variants within FSHD-associated genes [11,12]. The detection of DRAs requires specialized equipment and is labor-intensive, although more precise and automated approaches (such as molecular combing and single-molecule optical mapping) have been recently proposed as alternative methods. Overall, the availability of advanced workflows able to support the diagnosis in a time and cost-effective manner is of paramount importance. Considering that the DNA methylation status representative of *D4Z4* locus has been recognized as a hallmark of the disease, several research studies tested it as a possible diagnostic biomarker. In particular, a number of protocols have been proposed to assess the methylation status, although different CpG sites/regions, biological sources and variable sample sizes have been used [13,14,15,16,17,18,19,20,21]. Given these premises, mathylation analysis and Machine Learning (ML) pipelines were tested as possible methods to prioritize FSHD subjects for standard molecular testing and supporting the clinical diagnosis. An in-house protocol based on Bisulfite Sequencing (BSS) followed by Amplification Fragments Length Polymorphisms (AFLP) was employed to obtain methylation levels of single CpG sites from patients’ whole blood. Afterwards, statistical analyses and supervised ML methods were applied to assess the presence of reduced methylation profile compatible with FSHD and evaluate the overall method as a supporting tool in the diagnostic process of the disease.

## 2. Materials and Methods

### 2.1. Selection of the Cohort

The study involved two independent cohorts, namely a training group and a test set. Firstly, 133 FSHD subjects and 150 CTRL were employed as a training group for the development of the ML model. Furthermore, the test set including 52 subjects (namely, 27 FSHD and 25 CTRL) was subsequently analyzed for the testing of the ML model. The details concerning both study cohorts have been summarized in Table 1 and Supplementary Table S1A–C.

The FSHD subjects were recruited by expert neurologists from Fondazione Policlinico Gemelli IRCCS in collaboration with the Italian Union Foundation for the fight against muscular dystrophies (UILDM). Patients were evaluated on the basis of clinical and instrumental examinations [22,23,24,25]. 

The presence of DRA and likely pathogenic/pathogenic variants in FSHD genes was evaluated during the diagnostic workflow at the Genomic Medicine Laboratory-UILDM at the Santa Lucia Foundation IRCCS, with the purpose of considering either FSHD1, FSHD2 or FSHD1 + FSHD2 forms in the study. In particular, the molecular assessment of DRA was performed using PFGE and southern blotting followed by hybridization with specific probes P13-E11. The investigation of FSHD-associated variants has been performed by NGS analysis on an Illumina^®^ Next-Seq550 system and related kit. The FSHD patients (*n* = 133) of the training cohort displayed a number of RUs ranging from 1 RU to >10 RUs (16 patients with 1–3 RUs, 95 patients with 4–7 RUs, 10 patients with 8–10 RUs and 12 patients with >10 RUs). This cohort also included 15 patients with likely pathogenic/pathogenic variants within *SMCHD1* and *LRIF1*, of whom 11 FSHD1 + FSHD2 (5 ranging 4–7 RUs and 6 with 8–10 RUs) and 4 FSHD2 (>10 RUs). Concerning the test set (*n* = 27), 7 patients displayed 1–3 RUs, 18 had 4–7 RUs whereas 2 showed >10 RUs and likely pathogenic/pathogenic variants within *SMCHD1*. The selection of control subjects was based on the absence of any clinical sign suggestive of FSHD and were negative to DRA testing and to pathogenic variants in disease-associated genes.

### 2.2. Analysis of DNA Methylation and 4q Subtelomeric Variant Typing

**Figure 1:**
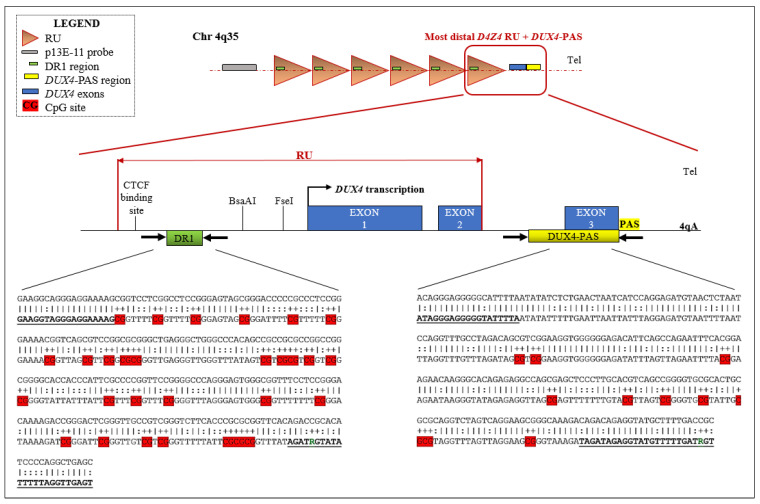
Schematic representation of the analyzed *D4Z4* regions together with their sequence within the *D4Z4* locus. The figure illustrates the locations of DR1 and *DUX4*-PAS target regions into the *D4Z4*. Moreover, the most distal *D4Z4* unit, encompassing the whole *DUX4* ORF is shown. For each target region, the corresponding sequence is reported. The upper line shows the non-converted genomic sequence, whereas the lower line displays the bisulfite converted sequence (as predicted by MethPrimer.com, accessed on 3 September 2022). Herein, the harbored specific CpG sites (29 CpGs for DR1 and 10 CpG for *DUX4*-PAS, respectively) are highlighted in red. The sequence of the employed primers is shown in bold and underlined. In particular, the modified nucleotides (R) in the primer sequences are shown. PAS: Polyadenylation Signal; Tel.: Telomere.

The methylation profiles of two regions of the *D4Z4* locus were assessed. In particular, the DR1 is located 1 Kb upstream of the *DUX4* ORF and harbors 29 CpG sites, whereas the *DUX4*-PAS is located within the most distal part of the array (including the PolyAdenilation Signal, PAS) and contains 10 CpG sites (Figure 1). Importantly, while the *DUX4*-PAS assay is specific for the 4q distal region (encompassing the more distal repeated unit), the DR1 region is located within each *D4Z4* RU on both chromosome 4 and 10.

The DNA from each patient has been subjected to methylation analysis according to an in-house protocol based on BSS and AFLP. DNA was extracted from whole blood by automated extraction using Blood kit Magpurix (Zinexts, Taipei, Taiwan). Successively, 500 ng of the extracted DNA was subjected to bisulfite conversion through EpiTect Bisulfite Kit (Qiagen, Germantown, MD, USA) according to the manufacturer’s instructions. The converted DNA was quantified by DS-11 FX Spectrophotometer (DeNovix, Wilmington, DE, USA) and 200 ng were amplified for the DR1 and *DUX4*-PAS regions using HotStarTaq Master Mix (Qiagen, Germantown, MD, USA) together with specific primers retrieved from Hartweck et al., 2013 and Calandra et al., 2016 [15,21], respectively. Of note, these primers were modified to improve the sequencing quality and the reliability of the obtained methylation levels. In particular, both primers were optimized by adding M13-Forward and -Reverse tails (Applied Biosystems) to improve the resolution of peaks during the sequencing. In addition, considering that the reverse primer specific for DR1 region (DR1-R) covers a single CpG site, it was modified in order to prevent the preferential amplification of unmethylated strand thus avoiding a possible underestimation of methylation levels. To this purpose, a mixture of DR1-R primers (Figure 1) that differ for one nucleotide (A or G) in the position corresponding to the CpG site was employed.

The resulting PCR products have been purified using Exonuclease I and Antartic Alkaline Phosphatase (Biolabs). Following quantification by means of Qubit 3.0 Fluorometer, purified amplicons have been subjected to SDS 2.2% pre-treatment at 98 °C for 5 min and then subjected to post-sequencing clean-up by means of Performa DTR Gel Filtration Cartridges according to manufacturer’s protocol. Afterward, the samples underwent Sanger sequencing using BigDye Terminator v3.1 Cycle Sequencing Kit (ThermoFisher Scientific, Waltham, MA, USA) followed by capillary electrophoresis on ABI Prism 3130× L Genetic Analyzer (Applied Biosystems). Then, samples have been run again using the AFLP protocol on the same instrument upon the addition of 0.5 μL of GeneScan-120 LIZ Dye Size Standard (Applied Biosystems). This step enabled the quantitative evaluation of methylation levels of all CpGs in both regions by analyzing the resulting data with the AFLP-specific analysis module in Gene Mapper software 5.0 (Applied Biosystems). Cytosines and Thymines peak heights have been compared to determine the percentage of methylated cytosine for each CpG site. By this method, the methylation patterns have been obtained and then employed for extensive biostatistical and computational analyses. 

The presence of 4qA subtelomeric allele was assessed for each converted DNA, since the successful amplification of *DUX4*-PAS was indicative of the presence of 4qA allele, whereas specific primers for the 4qB allele were retrieved from Calandra et al., 2016 [15] and used to set-up a specific PCR on converted DNA. The 4qB-positive samples were subjected to Sanger sequencing using BigDye Terminator v3.1 Cycle Sequencing Kit (ThermoFisher Scientific) for confirmation. Samples homozygous 4qB/4qB (i.e., negative to *DUX4*-PAS amplification) were not included among the samples’ cohorts.

### 2.3. Statistical Analysis

All of the statistical analysis was performed in R (v 4.1). Methylation levels in DR1 and *DUX4*-PAS regions were compared between groups using multiple one-way ANOVA for each comparison, namely: FSHD vs. CTRL; FSHD_low-RU_ vs. FSHD_high-RU_, FSHDvar+ vs. FSHDvar−. The obtained *p*-values (*p*) were corrected by False Discovery Rate (FDR) and deemed as statistically significant when FDR *p* < 0.05 (Supplementary Tables S2–S4). 

### 2.4. Machine Learning Pipeline for Classification

In order to test the discriminative power of the methylation levels related to the CpG sites of both *DUX4*-PAS and DR1, a supervised ML pipeline was implemented in R (v. 4.1.1) using the Caret package [26]. The ML pipeline follows IBM’s CRoss Industry Standard Process for Data Mining (CRISP-DM) to ensure the stability of results and replicability. The data frame used in our pipeline included all CpG sites and subjects’ year of birth. Missing values (~1%) were imputed using the bagged tree imputation method, where a bagged tree model is fitted for each predictor (as a function of all the others) to predict missing values [27].

From here on, a separated ML pipeline was implemented for each binary classification task: FSHD vs. CTRL, FSHD_low-RU_ vs. FSHD_high-RU_, FSHDvar+ vs. FSHDvar− (Supplementary Tables S5–S7).

#### 2.4.1. FSHD vs. CTRL

The training pipeline was implemented nesting several ML models and data preprocessing methods (Supplementary Table S5) on the training set. Importantly, the Leave-One-Out Cross-Validation (LOOCV) strategy was utilized for hyperparameters tuning. Only models known for their ability to manage intercorrelated predictors were included. Successively, the trained ML models were tested on an independent cohort used as a test set in order to select the final model achieving the highest accuracy metrics. The formula for the calculation of accuracy is reported in the File S1.

#### 2.4.2. FSHD_low-RU_ vs. FSHD_high-RU_ and FSHDvar+ vs. FSHDvar−

FSHD subjects in the FSHD_low-RU_ vs. FSHD_high-RU_ classification task were divided into “high-RU” subjects when RUs > 10 (*n* = 12) or “low-RU” when RUs ≤ 10 (*n* = 121). The same specifics from Section 2.4.1 were used in this training pipeline (Supplementary Table S6). Due to strong class imbalance, the final model was selected by comparing the achieved F1-Score from LOOCV. A training pipeline with the same specifics was used to classify FSHD subject in the FSHDvar+ (namely, patients with pathogenic/likely pathogenic variants in FSHD genes, *n* = 15) vs. FSHDvar− (patients negative to FSHD-related genetic variants, *n* = 118) classification task (Supplementary Table S7), and the final model was selected based on the F1-Score reported during LOOCV. The formula for the calculation of F1-Score is reported in the File S1.

## 3. Results

### 3.1. Statistical Analysis

The study involved two cohorts (namely the training cohort and the test set) as previously described (Table 1, Supplementary Table S1A–C). All the subjects analyzed in the study were characterized by at least one 4qA subtelomeric allele. The training cohort displayed the following 4q genotype distribution, FSHD: 52% AA, 48% AB; CTRL: 34% AA, 66% AB. The 4q genotype distribution in the test set was FSHD: 48% AA, 52% AB; CTRL: 24% AA, 76% AB. 

**Figure 2:**
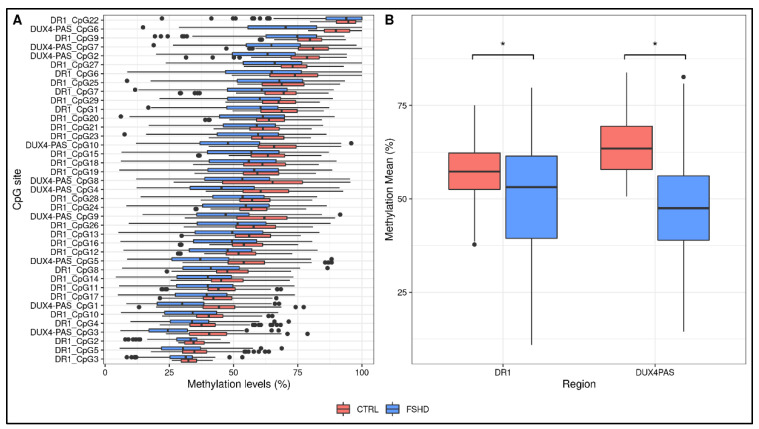
Descriptive plots of the analyses performed on the study cohorts. (**A**) A boxplot showing the methylation levels of each CpG site within DR1 and *DUX4*-PAS regions related to the training group (FSHD *n* = 133, CTRL *n* = 150). The lower and upper hinges correspond to the 25th and 75th percentiles of the distribution. The whiskers extend from the hinge to the largest value no further than ±1.5 × IQR upper whisker extends from the hinge to the largest value no further than 1.5 × IQR from the hinge (where IQR is the inter-quartile range, or distance between the first and third quartiles). The lower whisker extends from the hinge to the smallest value at most 1.5 × IQR of the hinge. Data beyond the end of the whiskers are called “outlying” points and are plotted individually. (**B**) The average methylation levels are different between groups for both DR1 and *DUX4*-PAS regions. * *p* < 0.05.

DNA methylation levels for each CpG site within DR1 and *DUX4*-PAS regions were obtained for each sample. The multiple FDR-corrected ANOVA revealed that all CpG sites harbored by FSHD subjects showed significantly reduced methylation (i.e., hypomethylation) compared to the controls (FDR *p* < 0.001, Figure 2A, Supplementary Table S2). Accordingly, the average methylation levels of the whole regions were significantly lower (DR1 FDR *p* = 2 × 10^−8^, *DUX*4-PAS FDR *p* = 6 × 10^−29^, Figure 2B) in patients compared to the controls.

As reported in Supplementary Table S2, the analysis revealed that CpG sites showed variable significance values, suggesting that single CpG sites differentially contribute to the methylation pattern of the *D4Z4*. 

**Figure 3:**
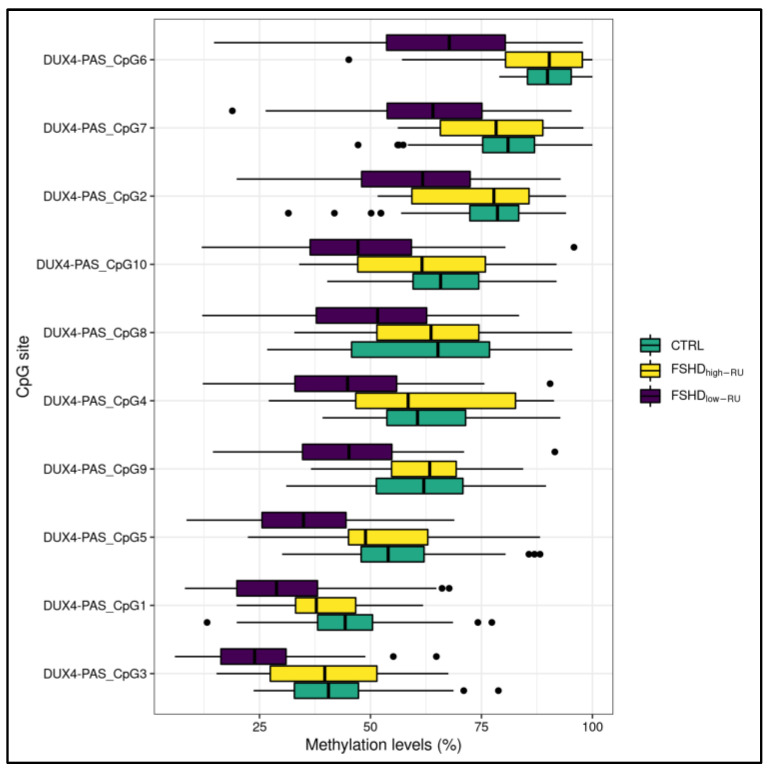
A boxplot showing the methylation levels related to *DUX4*-PAS CpG sites of FSHD_low-RU_ (*n* = 121), FSHD_high-RU_ (*n* = 12) and CTRL subjects. The lower and upper hinges correspond to the 25th and 75th percentiles of the distribution. The whiskers extend from the hinge to the largest value no further than ±1.5 × IQR (where IQR is the inter-quartile range, namely distance between the first and third quartiles). Data points beyond the whiskers are plotted individually.

The methylation levels related to DR1 and *DUX4*-PAS regions were compared between FSHD subjects with a high (>10 RU, namely FSHD_high-RU_) and low (≤10 RU, namely FSHD_low-RU_) range of RU number. As a result, FSHD_low-RU_ patients displayed significant (0.01 < FDR *p* < 0.05) hypomethylation levels at nine CpG sites within the *DUX4*-PAS region (Figure 3, Supplementary Table S3).

**Figure 4:**
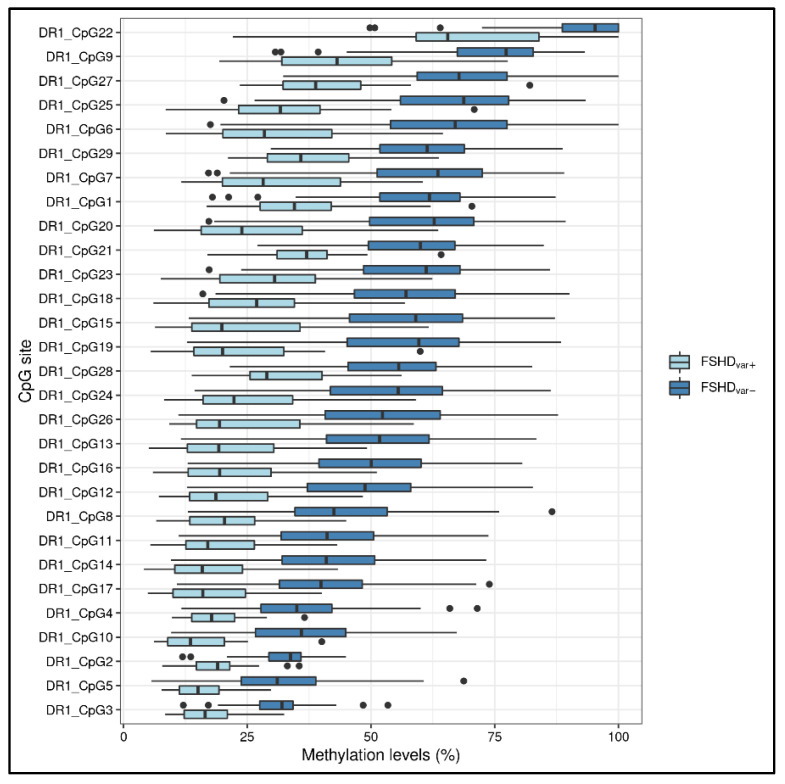
A boxplot showing the methylation levels related to the DR1 CpG sites of FSHDvar+ (*n* = 15) and FSHDvar− (*n* = 118). The lower and upper hinges correspond to the 25th and 75th percentiles of the distribution. The whiskers extend from the hinge to the largest value no further than ±1.5 × IQR (where IQR is the inter-quartile range, namely distance between the first and third quartiles). Data points beyond the whiskers are plotted individually.

Moreover, the methylation levels were also compared in patients harboring likely pathogenic/pathogenic variants in FSHD genes (*SMCHD1*, *LRIF1*) with respect to the other patients (namely, FSHDvar+ vs. FSHDvar− comparison). Of note, 11 out of the 15 patients were characterized by a DRA ≤ 10 RUs. As a result, all the CpG sites within the DR1 displayed significantly lower methylation levels (Figure 4) in these subjects (5.29 × 10^−6^ < FDR *p* < 3.11 × 10^−4^, Supplementary Table S4), whereas only one site within *DUX4*-PAS (namely, CpG4) appeared to show statistically significant differences (FDR *p* = 0.008).

### 3.2. Development of a ML-Based Classifier for the Discrimination of FSHD Subjects

**Figure 5:**
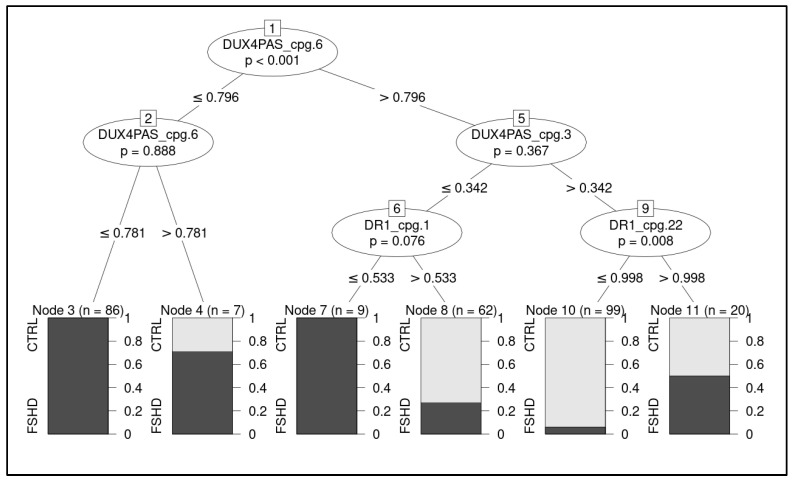
Illustration of the decision tree showing the hierarchical order of decisions to discriminate between groups. The considered CpG sites are highlighted with relative decision thresholds based on methylation levels. The boxes report predicted class, relative proportion of subjects belonging to the group (CTRL and FSHD, respectively) and number of classified subjects per node. The *p*-values refers to permutation test of the model.

A ML pipeline was employed to build a classification model able to discriminate FSHD subjects from CTRL. The most accurate classifier fitted on raw data (retrieved from the training set of subjects) resulted to be the conditional inference tree (Figure 5, Supplementary Table S5). 

**Figure 6:**
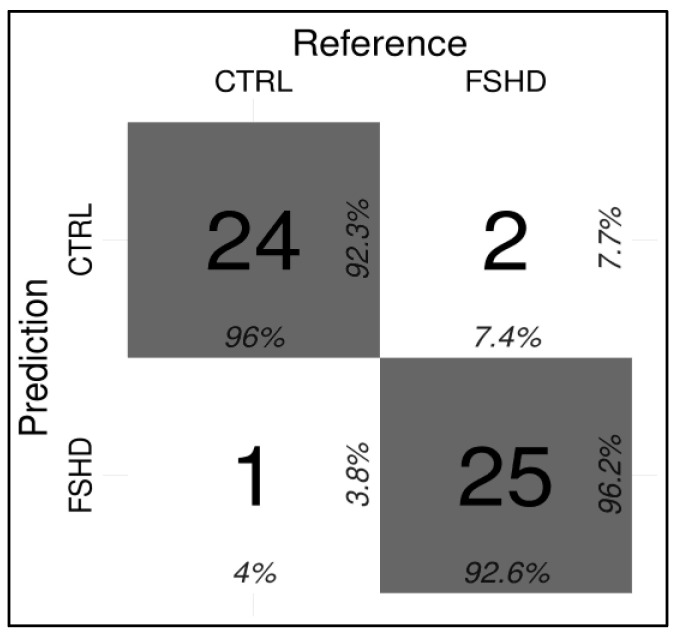
Confusion matrix indicating the correct and incorrect predictions performed by the ML classifier (Conditional Inference tree) for the FSHD vs. CTRL comparison.

On the test cohort, the model achieved 0.94 accuracy, 0.93 AU-ROC, 0.93 sensitivity, and 0.96 specificity, correctly identifying 25/27 FSHD subjects and 24/25 CTRL (Figure 6). 

In particular, the methylation levels related to four CpG sites, namely *DUX4*-PAS_CpG6, *DUX4*-PAS_CpG3, DR1_CpG1 and DR1_CpG22, were identified as the most relevant for the discrimination of FSHD subjects and were used in the decision tree (Figure 5).

The conditional inference tree model was also tested on average methylation levels of DR1 and *DUX4*-PAS, although the obtained metrics (accuracy: 0.87, AU-ROC: 0.79, sensitivity: 0.85, specificity: 0.88) provided lower performance rates with respect to the model fitted on single CpG sites. This result indicates that the methylation levels of single CpG sites are more informative than region means. 

**Figure 7:**
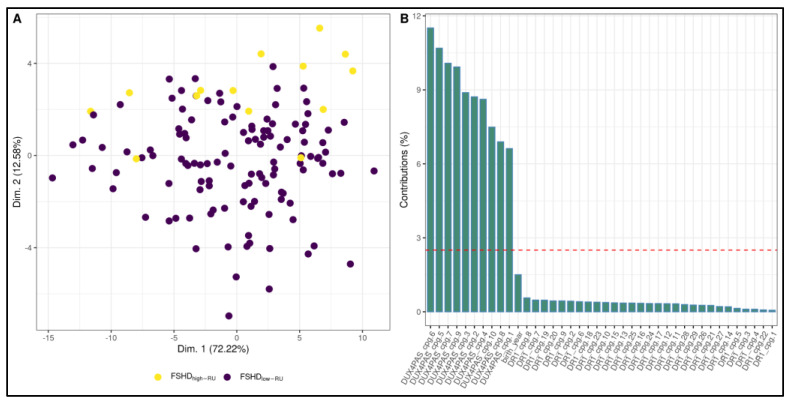
Random forest model for the discrimination between FSHD_low-RU_ and FSHD_high-RU_ subjects. (**A**) A PCA plot highlighting slight separation between FSHD_low-RU_ and FSHD_high-RU_ groups. In particular, the largest separation appears on Dimension 2. (**B**) Suggestively, variable contributions to Dimension 2 are mostly from *DUX4*-PAS CpG sites. The most important variable for the selected Random Forest (fitted on the PCA dimensions) is indeed Dimension 2.

In addition, the ML pipeline was used to test the ability of methylation data to discriminate FSHD_low-RU_ and FSHD_high-RU_ subjects. A random forest fitted on the PCA (Figure 7A) of the data was selected as classification model (Supplementary Table S6). The model obtained 0.81 accuracy, 0.82 AU-ROC, 0.86 sensitivity and 0.81 specificity. Variable importance confirmed the pivotal role of *DUX4*-PAS region in differentiating FSHD_low-RU_ vs. FSHD_high-RU_ (Figure 7B). 

Furthermore, the ML pipeline was used to classify FSHD individuals harboring likely pathogenic/pathogenic variants with respect to negative subjects (FSHDvar+ vs. FSHDvar−). In this case, a conditional inference tree model fitted on data with the exponential transformation was selected as the best classifier (Supplementary Table S7). In particular, the model achieved 0.90 accuracy, 0.88 AU-ROC, 0.80 sensitivity and 0.92 specificity and identified the DR1_CpG3 as the most discriminating site, considering a threshold of methylation levels of ≤0.37.

## 4. Discussion

FSHD is characterized by a strong epigenetic component marked by a *D4Z4* hypomethylation status that is a necessary condition for DUX4 toxic activation and, subsequently, for disease manifestation [28]. Therefore, the assessment of *D4Z4* methylation patterns can support the clinical and molecular diagnosis in the near future, especially if performed on easily-accessible sources without the need of invasive procedures. Of note, previous studies evaluated the absence of differences between DNA methylation profiles of the *D4Z4* locus related to muscular tissues, blood cells and saliva [29,30]. 

Here, an optimized technical protocol combined with specific ML models is proposed as a tool to discriminate FSHD patients from controls. In particular, the methylation levels of the DR1 and *DUX4*-PAS regions (Figure 1) were measured in a first cohort (namely, the training cohort) including a large number of patients (*n* = 133) and compared with CTRL (*n* = 150). As expected, the methylation levels were found significantly reduced in FSHD compared to CTRL subjects in each CpG site of both regions (Figure 2). Statistical analysis revealed variable significant values for single CpG sites, suggesting that each of them shows a differential discriminative value. Therefore, the obtained data were then used to train a ML model (conditional inference tree) for the identification of FSHD subjects. In particular, this model was evaluated on the test set of 52 subjects that were subsequently analyzed to calculate the accuracy metrics and highlight the most relevant CpG sites for discriminating FSHD subjects. This analysis pointed out four single CpG sites (namely, *DUX4-PAS*_CpG6, *DUX4-PAS*_CpG3, *DR1*_CpG1 and *DR1*_CpG22) as the most relevant for FSHD subjects’ discrimination (Figure 5 and Figure 6). Considering the high performance metrics (accuracy: 0.94, sensitivity: 0.93, specificity: 0.96, Figure 6) achieved by the developed classifier, this approach appears as a powerful tool supporting clinical and molecular diagnosis. 

As shown in Figure 6, the testing of the model on the test set showed three misclassified subjects. In fact, two samples (referred to as sample ID16 and ID27 in Supplementary Table S1C) belonging to the FSHD group were classified as non-FSHD and consistently, displayed higher methylation levels. It is important to point out that for a proper interpretation of these cases we need to consider other information such as the 4qA/4qA subtelomeric configuration. 

Indeed, both samples referred to patients harboring a 4qA/4qA genotype, which could overestimate the methylation levels due to the fact that the assay would detect the methylation levels of both alleles, in contrast to subjects with a single copy of 4qA that would provide a more precise measure. Of note, this similar issue has also been highlighted in the recent study by Erdmann et al., 2022 [19]. 

This issue raises the need for performing a study including a larger cohort of 4qA/4qA and 4qA/4qB samples, in order to account for this data in the classification of FSHD subjects. Nevertheless, it is important to remark that the model was able to correctly identify the other patients (*n* = 10) carrying a 4qA/4qA and all the patients (*n* = 15) with 4qA/4qB genotype of the test set. The third misclassified patient (namely ID44 in Supplementary Table S1C) belonged to CTRL group, although he showed lower methylation levels than expected. Indeed, the subject was referred to our center as a non-affected subject, suggesting thereby a possible asymptomatic condition. Considering his positive family history for FSHD, this subject is currently under clinical monitoring and will be subjected to additional genetic analyses. This result further suggests a potential application of methylation analysis for identifying asymptomatic subjects which could benefit of a specific follow-up over time. However, a larger cohort of similar patients is needed to confirm this hypothesis.

Moreover, the application of ML approaches highlighted that the methylation levels of single CpG sites are more informative than region means. Supporting this data, the testing of the model on average methylation levels of DR1 and *DUX4*-PAS showed lower performance rates (accuracy: 0.87, AU-ROC: 0.79, sensitivity: 0.85, specificity: 0.88) with respect to the model fitted on single CpG sites. This result indicates that the methylation levels referred to the single CpG sites should be preferred for the accurate classification of FSHD subjects. Indeed, various studies investigated the association of reduced *D4Z4* methylation levels with the disease, though reporting variable results depending on different sample sizes, employed methodologies (BSS, long read sequencing, antibody-based methods and utilization of methylation-sensitive restriction enzymes) and analyzed region/CpG sites (whole *D4Z4* unit, 5′ *DUX4*-ORF, distal region of 4q35) [13,14,15,16,17,18,21,29]. On this subject, the study by Erdmann et al., 2022 performed an evaluation of *D4Z4* methylation in a diagnostic workflow aimed at enhancing the interpretation of disease manifestations [19]. Importantly, the study is based on a BSS-NGS approach, focusing their attention on the 4q distal region and the entire repeated unit. By this way, they reported a reduced average methylation of the detected CpG sites in FSHD subjects, which is in accordance with our data. Moreover, they found the association of these methylation profiles with the disease severity, as also reported by previous studies, and propose the application of DNA methylation into the diagnostic workflow [19]. 

Furthermore, a recent study by Hiramuki et al., 2022 tested a long-read sequencing-based approach, which allowed the authors to simultaneously analyze the *D4Z4* methylation and size in FSHD patients. Additionally in this case, the authors found reduced global *D4Z4* methylation levels in FSHD samples and provide precise insights into the pathological epigenetic status of *D4Z4* locus [20]. Indeed, our results are consistent with all the aforementioned data and further support the applicability of DNA methylation assessment and 4q haplotyping to prioritize or exclude patients for FSHD diagnostic testing. Remarkably, most of previous and recent studies focused on average methylation levels, whereas the present study took advantage of a fine analysis and ML pipelines to highlight the higher discriminative power of single CpG sites rather than region means. If these results will be validated in larger studies, they will pave the way for more targeted, rapid and less expensive assays for methylation assessment.

FSHD subjects with a DRA ≤ 10 RUs displayed lower methylation levels within *DUX4*-PAS-related CpG sites (Figure 3) with respect to subjects with >10 RUs. In particular, the CpG sites located within *DUX4*-PAS were more informative for this comparison (Figure 7). This evidence is in line with other literature data showing a correlation between the methylation levels of *DUX4*-PAS_CpG6 and the RUs number [15]. In this case, the model achieved an accuracy of 0.81 in identifying patients with a DRA. In particular, this accuracy value may also reflect the variable penetrance of DRA [31] as well as the possible presence of *D4Z4* contraction (4–8 RUs) in ~3% of healthy individuals [32].

The ability of methylation patterns to suggest the presence of detrimental variants within FSHD-associated genes was also evaluated. In line with other studies, the most striking hypomethylation levels were found in DR1 (Figure 4) [7,21,33]. Although DR1 assay is not specific for the 4q copies and these regions are present also on chromosome 10, our data showed that it did not affect the detectability of hypomethylation profiles, that are heavily reduced in presence of pathogenic variants in FSHD genes. This finding is in accordance with previous studies reporting similar observations [17,21]. In addition, the application of long read sequencing found comparable reduced *D4Z4* methylation levels for both 4q and 10q in FSHD2 patients [20]. 

The conditional inference tree fitted on data with the exponential transformation displayed the highest metrics (accuracy: 0.90, sensitivity: 0.80, specificity: 0.92) in discriminating patients with likely pathogenic/pathogenic variants (namely, FSHDvar+ subjects). In particular, the model utilizes the DR1_CpG3 as the most discriminating site and considers a methylation level threshold ≤0.37 for classifying FSHDvar+ subjects. In our cohort, 11 FSHD patients out of a total of 15 displayed both a DRA and likely pathogenic/pathogenic variants within FSHD genes (namely, *SMCHD1* and *LRIF1*), consistent with the presence of a compound form of disease (FSHD1 + FSHD2). The remaining four FSHD2 samples showed comparable methylation levels with those displayed by the other FSHD1 + FSHD2 patients. This finding further suggests that patients with likely pathogenic/pathogenic variants may be correctly identified using methylation data independently from the presence of DRA. Moreover, the presence of patients with FSHD1 + FSHD2 forms of disease further confirms that FSHD1 and FSHD2 are not mutually exclusive, because DRA and pathogenic variants may co-occur as a part of wider spectrum of disease [1,34].

The present study took advantage of the optimization of the molecular protocol used for measuring the levels of methylation and the application of ML for enhancing the sensibility and specificity of the assay. Other advantages related to the presented method include its rapidity (~72 h), accessibility (~15 €/sample), easiness and health-safety (no use of toxic reagents) compared to other methylation assays. 

Importantly, the application of methylation analysis to subjects with a clinical suspicion of FSHD could provide specialists with preliminary evidence to be confirmed by traditional DRA assessment. Furthermore, the use of ML pipelines is expected to promote the standardization of non-automated technical procedures such as methylation analysis. 

In conclusion, the application of methylation analysis and ML was able to successfully distinguish FSHD patients from controls, providing additional evidence for DNA methylation as a powerful disease biomarker to be exploited for a rapid and reliable prioritization of FSHD subjects to be confirmed by standard testing (*D4Z4* sizing, research for FSHD-associated variants). Moreover, our study is in line with the recent application of ML for enhancing the clinical diagnosis and decision-making performance in several medical fields, including oncology, cardiology, ophthalmology and neurology [35,36,37,38]. In addition, ML-based methods have also been tested for fostering the research of molecular disease biomarkers in different diseases and phenotypes, including neuromuscular disorders [25,39,40]. On this subject, ML models allowed identifying single CpG sites in *DUX4*-PAS and DR1, enabling an accurate discrimination of FSHD subjects (either FSHD1, FSHD2 or compound forms). 

Finally, multicentric and multidisciplinary studies on larger cohorts are required to confirm the results of the presented approach and to test its utility in a clinical routine use.

## Figures and Tables

**Table 1:** Descriptive statistics of cohorts’ demographics.

Condition	Cohort	*n*	Mean Age (±SD)	F:M Ratio
FSHD	Training group	133	51.4 (±17.6)	45:55
CTRL	Training group	150	55.7 (±15.8)	36:64
FSHD	Test set	27	56.0 (±16.7)	45:55
CTRL	Test set	25	50.0 (±14.7)	52:48

## Data Availability

All data generated in this manuscript are included within the manuscript. The machine learning-based methods which have been tested and the summary results of the performed ANOVA are reported in the File S1. Additional information is available on request to the authors, providing that they are used for noncommercial purposes.

## Supplementary Materials

The following supporting information can be downloaded at: https://www.mdpi.com/article/10.3390/cells11244114/s1. Table S1: (**A**) features of the FSHD training group. The demographic features and molecular data related to the 133 FSHD subjects of the training group are reported for each patient; (**B**) features of the CTRL training group. The demographic features and molecular data related to the 150 CTRL subjects of the training group are reported. “D4Z4 size” and “Pathogenic/likely-pathogenic variant” columns are not reported since these subjects were negative to DRA testing and pathogenic variants in disease-associated genes; (**C**) features of the CTRL training group. The demographic features and molecular data related to the 150 CTRL subjects of the training group are reported. “D4Z4 size” and “Pathogenic/likely-pathogenic variant” columns are not reported since these subjects were negative to DRA testing and pathogenic variants in disease-associated genes. Table S2: summary of the ANOVA tests for FSHD vs. CTRL. Column “y” reports the tested variable; “df” reports the degrees of freedom of the ANOVA; “sumsq”, “meansq”, “statistic” and “p.value” columns report ANOVA summary information; “sig.p” column indicates if the corresponding p.value is significant (*p* < 0.05) or not; “fdr” reports the FDR-corrected p.value, and “sig.fdr” indicates if the corresponding fdr is significant (fdr < 0.05) or not. Table S3: summary for the ANOVA tests performed for FSHDlow-RU vs. FSHDhigh-RU. Column “y” reports the tested variable; “df” reports the degrees of freedom of the ANOVA; “sumsq”, “meansq”, “statistic” and “p.value” columns report ANOVA summary information; “sig.p” column indicates if the corresponding p.value is significant (*p* < 0.05) or not; “fdr” reports the FDR-corrected p.value, and “sig.fdr” indicates if the corresponding fdr is significant (fdr < 0.05) or not. Table S4: summary for the ANOVA tests performed for FSHDvar+ vs. FSHDvar-. Column “y” reports the tested variable; “df” reports the degrees of freedom of the ANOVA; “sumsq”, “meansq”, “statistic” and “p.value” columns report ANOVA summary information; “sig.p” column indicates if the corresponding p.value is significant (*p* < 0.05) or not; “fdr” reports the FDR-corrected p.value, and “sig.fdr” indicates if the corresponding fdr is significant (fdr < 0.05) or not. Table S5: ML model evaluation metrics for FSHD vs. CTRL. Columns 1 reports the evaluated metric. The last two columns specify the data preprocessing strategy used and the ML method trained, respectively. Prep legend: NTH = no preprocessing (raw data); BOX = BoxCox transformation; YEO =YeoJohnson transformation; PWR = exponential transformation; CTR = center and scale; PCA =Principal Component Analysis (5 dimensions); SPA = Spatial Sign transformation. Method legend can be consulted at https://topepo.github.io/caret/available-models.html (Accessed on 1 April 2022). Table S6: ML model evaluation metrics for FSHDhigh-RU vs. FSHDlow-RU. Columns 1 reports the evaluated metric. The last two columns specify the data preprocessing strategy used and the ML method trained, respectively. Prep legend: NTH = no preprocessing (raw data); BOX = BoxCox transformation; YEO =YeoJohnson transformation; PWR = exponential transformation; CTR = center and scale; PCA = Principal Component Analysis (5 dimensions); SPA = Spatial Sign transformation. Method legend can be consulted at https://topepo.github.io/caret/available-models.html (Accessed on 1 April 2022). Table S7: ML model evaluation metrics for FSHDvar+ vs. FSHDvar-Columns 1 reports the evaluated metric. The last two columns specify the data preprocessing strategy used and the ML method trained, respectively. Prep legend: NTH = no preprocessing (raw data); BOX = BoxCox transformation; YEO = YeoJohnson transformation; PWR = exponential transformation; CTR = center and scale; PCA = Principal Component Analysis (5 dimensions); SPA = Spatial Sign transformation. Method legend can be consulted at https://topepo.github.io/caret/available-models.html (Accessed on 1 April 2022). File S1: Supplementary Methods.
