# Supplementary material for: D4Z4 Methylation Levels Combined with a Machine Learning Pipeline Highlight Single CpG Sites as Discriminating Biomarkers for FSHD Patients
Source: Cells. 2022 Dec 18;11(24):4114. doi: 10.3390/cells11244114 (PMC9777431; doi:10.3390/cells11244114)
Supplement: Supplementary file 1 [file cells-11-04114-s001.zip › cells-1969223-supplementary-File S1.pdf]

Supplementary Methods

The accuracy score was defined as follows:

$$Accuracy = \frac{\#\ of\ correct\ predictions}{\#\ of\ total\ predictions}$$

The F1-Score was defined as the harmonic mean of Precision and Recall:

$$Precision = \frac{\#\ of\ True\ Positives}{\#\ of\ True\ Positives\ +\ \#\ of\ False\ Positives}$$

$$Recall = \frac{\#\ of\ True\ Positives}{\#\ of\ True\ Positives\ +\ \#\ of\ False\ Negatives}$$

$$F1 = 2 \times \frac{Precision\ \times\ Recall}{Precision\ +\ Recall}$$
